# Risk model of liquid–liquid phase separation‐related genes reveals the prognosis and tumor microenvironment characteristics of colorectal cancer

**DOI:** 10.1002/ccs3.70054

**Published:** 2025-11-03

**Authors:** Hui Liu, Ziwen Chen, Jie Hao, Ziyi Dong, Yaoyang Guo, Minghan Qiu, Xipeng Zhang, Ming Gao, Haiyang Zhang, Mingqing Zhang

**Affiliations:** ^1^ Tianjin Institute of Coloproctology Tianjin Union Medical Center The First Affiliated Hospital of Nankai University Nankai University Tianjin China; ^2^ Key Laboratory of Cancer Prevention and Therapy Tianjin Medical University Cancer Institute and Hospital National Clinical Research Center for Cancer Tianjin's Clinical Research Center for Cancer Tianjin China

**Keywords:** colorectal cancer, drug sensitivity, immune infiltration, liquid–liquid phase separation, prognostic model

## Abstract

Colorectal cancer (CRC) progression involves liquid–liquid phase separation (LLPS), but its prognostic significance remains unexplored. Using The Cancer Genome Atlas transcriptomic data, we developed an LLPS‐based risk model that outperformed traditional clustering methods. High‐risk patients exhibited worse outcomes, correlating with higher tumor mutational burden and reduced natural killer/T‐cell infiltration, yet increased predicted response to immune checkpoint blockade. Drug sensitivity analysis suggested therapeutic efficacy of Entinostat and 5‐fluorouracil in this subgroup. Five pivotal genes (ASXL1, DDX21, HNRNPA1L2, TACC3, and TRIM28) were identified as LLPS‐driven regulators of CRC progression, mechanistically linking phase separation to epigenetic dysregulation, aberrant RNA splicing, and metabolic reprogramming. Our study provides the first LLPS‐associated prognostic framework for CRC, offering both a risk stratification tool and actionable therapeutic insights. The findings highlight LLPS as a critical molecular organizer in CRC pathogenesis and a potential target for precision oncology approaches.

## Graphical abstract

A prognostic framework for colorectal cancer based on liquid–liquid phase separation (LLPS) genes stratifies patients by survival and tumor microenvironment. The pivotal gene signature—encompassing ASXL1, DDX21, HNRNPA1L2, TACC3, and TRIM28—is functionally linked to epigenetic and splicing dysregulation, providing independent risk assessment and therapeutic insights.

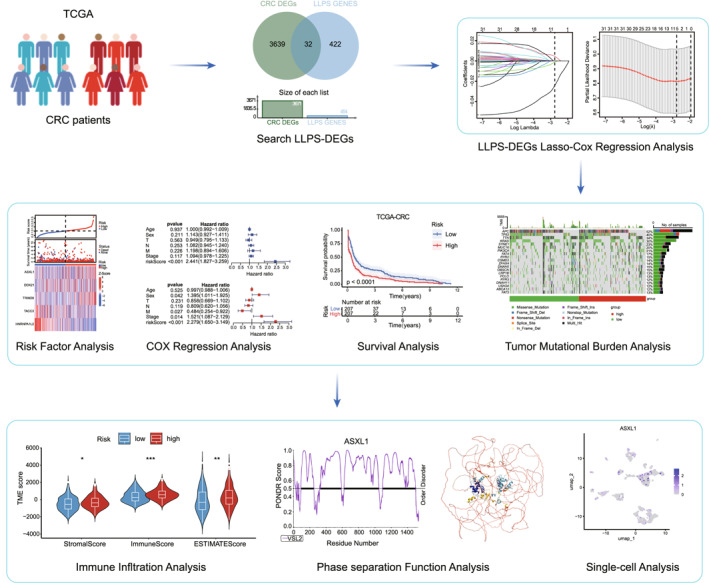

## 1 INTRODUCTION

Colorectal cancer (CRC) is one of the most common malignancies of the digestive system worldwide.^1^, ^2^ Global cancer data from 2020 show that CRC is the third most common malignancy and the second leading cause of cancer‐related death worldwide, resulting in over 900,000 deaths each year.^3^ Despite available therapies such as surgery, chemotherapy, radiotherapy, and immunotherapy, CRC is often characterized by rapid progression.^4^ Although it seems that there are many treatment options available for patients with CRC, in reality, the treatments available for patients with advanced metastatic CRC are still very limited, and their overall survival (OS) time and quality of life are poorer than those without metastasis.^5^, ^6^ CRC has an insidious onset. By the time patients show symptoms, it is often already at an advanced stage. Endoscopy is an excellent screening method for CRC. It has a high detection rate for precancerous lesions and early tumors, and can identify and reduce the incidence and mortality of CRC. However, this invasive examination is time‐consuming and costly, making it difficult to popularize in developing countries.^7^ Therefore, understanding the pathogenesis of CRC, developing early diagnostic techniques, and seeking more accurate early diagnostic indicators for CRC have become the focus of current research work.

Phase separation is an important discovery in the field of life sciences in recent years. It forms membrane‐free organelles (such as nucleoli, stress particles, etc.) through dynamic movement and regulates gene expression, signal transduction, and cell functions in time and space.^8^, ^9^ Studies have shown that abnormal phase separation is closely related to tumorigenesis and development and has been proven to be involved in a variety of intracellular biological processes.^10^, ^11^, ^12^ Liquid–liquid phase separation (LLPS) achieves high expression of related oncogenes by regulating super enhancer‐related proteins MED1 and BRD4 or transcriptional co‐activator (TAZ) yes‐related protein and its paracrine homolog, TAZ containing PDZ binding motifs.^9^, ^13^ The condensation state of 53BP1 can be modulated by various long noncoding RNAs (lncRNAs), thereby influencing DNA damage repair at double‐strand break sites.^14^ In addition, the binding of cyclic GMP‐AMP synthase to double‐stranded DNA induces LLPS of cGAS–DNA condensates, which creates a unique biochemical compartment that facilitates efficient DNA sensing and downstream pathway activation. Similarly, the phase separation of regulatory subunit Iα (RIα) has been shown to directly participate in the regulation of the cAMP/PKA signaling pathway.^15^ Furthermore, phase separation provides a new explanation for tumor metabolic heterogeneity. Metabolic enzymes (such as ACLY, PKM2) can form metabolic chambers through phase separation to dynamically regulate the local concentrations of carcinogenic metabolites (such as acetyl‐COA and α‐KG), thereby affecting histone modification and cell proliferation.^16^ This phase separation coupling of the “epigenetic‐metabolism‐splicing” network may become a breakthrough for targeted therapy of CRC. Focusing on exploring how it integrates the multi‐level regulatory network to drive malignant transformation of tumors is expected to provide a theoretical basis for the development of precise strategies targeting phase separation. In the existing studies, the research on the expression pattern, predictive value and molecular function of LLPS in CRC is limited. Therefore, this study aims to develop and confirm a predictive model for LLPS‐related genes in CRC and evaluate the potential role of LLPS in CRC. According to the prediction model, the cancer genome atlas (TCGA) data were divided into two groups of high and low risks. Subsequently, we conducted correlation analyses on copy number changes, enrichment pathway analysis, immune checkpoint degree, immune cell infiltration and drug sensitivity in the high‐ and low‐risk groups. In addition, molecular functional analyses were conducted on five prognostic prediction genes. This study can provide some potential targets for the precise treatment of CRC and offer certain references for the research of CRC LLPS.

## 2 MATERIALS AND METHODS

### 2.1 Transcriptome data download and processing

RNA sequencing transcriptome data and related clinical data were obtained from TCGA (https://portal.gdc.cancer.gov/). CRC cases and adjacent normal tissue samples were included in the analysis. Clinical data were collected for age, gender, stage and tumor, lymph node, and metastasis stage (Table S1). We identified 454 phase separation‐related genes through the phase separation protein database (PhaSepDB). As a validation set, we used the comprehensive database GSE72968, DOI: 10.1016/j.ejca.2017.02.003 from gene expression of a dataset. Table S2 represents the phase separation‐related genes used in this study.

### 2.2 Screen the DEGs related to LLPS

The R package “limma” was used to screen for differentially expressed genes (DEGs) in the CRC cohort. The screening conditions require that the *p* value should be <0.05 and |log2FC| > 1. Subsequently, we extracted the expression data of 32 DEGs related to LLPS (LLPS‐DEGs) from the DEGs in the CRC cohort for subsequent analysis. Table S3 provides the differential gene matrices between the normal group and the tumor group, and Table S4 provides 32 differential gene expression matrices related to LLPS.

### 2.3 Enrichment analysis

Use the R package “clusterProfiler” for gene ontology (GO) and kyoto encyclopedia of genes and genomes (KEGG) enrichment analysis. Next, we used a ranked genelist to gene sete enrichment analysis (GSEA) algorithm with default parameters. In order to find out which significant pathway (*q* < 0.25), the specific condition refers to HALLMARK and C2 from MsigDB database (https://www.gsea‐msigdb.org/gsea/msigdb).

### 2.4 Construction of prognostic risk model

Based on the differential expression analysis results of LLPS‐related genes in CRC, we used the R package “glmnet” for LASSO analysis and identified the five most predictive characteristic genes, including ASXL1, DDX21, HNRNPA1L2, TACC3, and TRIM28. Subsequently, the risk score of each CRC patient was calculated according to the following formula:

Riskscore=ΣexpGenei×βi.

In the formula, “expGenei” is the relative expression of prognostic model genes, and “*β*i” is the regression coefficient. According to the median risk score, CRC patients were divided into two groups. Then, the Kaplan–Meier (KM) curve was used to show the difference in OS between the two groups. The R package “timeROC” was used to display the receiver operating characteristic (ROC) curve to evaluate the prognostic ability of the prognostic risk model. Finally, the stability of the prognostic risk model was investigated in the validation dataset GSE72968.

### 2.5 Evaluation of the prognostic risk model

To determine whether clinicopathological characteristics and risk scores were independent predictors of CRC patients, we conducted univariate and multivariate Cox analyses. The nomogram was constructed using the R package “rm” to predict the survival probability based on independent prognostic criteria. The calibration curve is adopted to verify whether the nomogram has a good predictive ability. The closer the calibration curve is to the diagonal, the more accurate the prediction will be.

### 2.6 CMS classification

The consensus molecular subtypes (CMS) classification of each TCGA‐CRC sample was performed using the R package “CMSclassifier.” The classifier was applied to the log2‐CPM expression matrix using the recommended random forest method. Samples were assigned to one of the four consensus molecular subtypes (CMS1–CMS4) based on the highest‐class probability output by the classifier. Samples with a prediction probability <0.5 were deemed low‐confidence and were excluded from subsequent subtype‐specific analyses.

### 2.7 Consensus clustering based on LLPS‐related genes

Consensus clustering is an unsupervised clustering method that can divide samples into subtypes based on different datasets, thereby discovering new disease subtypes. The CRC queue was divided into two different subgroups using the R package “ConsensusClusterPlus.” The R package “timeROC” was used to display the receiver ROC curve to evaluate the prognostic ability of the prognostic risk model grouping and the prognostic ability of the consensus clustering grouping.

### 2.8 Tumor mutational burden (TME) analysis in different risk models

The tumor cell mutation information was downloaded from the TCGA database, and the number of mutations between the two risk groups of CRC patients was analyzed using the R package “maftools.”

### 2.9 Immune‐related functional analysis in different risk models

The proportion of immune cell type infiltration was calculated using the R package “cibersort.” The correlations of matrix, immunity, estimated score, and tumor purity between the high‐ and low‐risk groups were compared using the R package “estimate.” Subsequently, in order to understand the differences between the high‐ and low‐risk groups, the expressions of immune checkpoints and HLA family genes were analyzed.

### 2.10 Drug sensitivity analysis in different risk models

To explore the changes in drug sensitivity among risk groups, we used the R packages “pRRophetic” and “ggpubr” to calculate the IC50 values of commonly used immunotherapy drugs in cancer treatment.

### 2.11 Single‐cell sequencing analysis

Single‐celled GSE232525 datasets, DOI: 10.3389/fonc.2023.1219642 were downloaded from the GEO (http://www.ncbi.nlm.nih.gov/geo).. It contains two tumor samples of CRC. The scRNA‐seq data were analyzed using the “Seurat” software package of R software. First, the data is standardized. The “FindVariableFeatures” function is used to filter out the first 2000 highly variable genes. The “RunPCA” function was used to conduct principal component analysis on 2000 genes. The “Harmony” function removes the batch effect for different samples and uses uniform manifold approximation and projection for reduction and cluster identification. Use the R package “SingleR” to annotate different cell clusters.

### 2.12 Cell lines, cell culture, and cell transfection

The human CRC cell lines HCT116 and LoVo were derived from the Chinese Academy of Sciences. HCT116 and LoVo cells were cultured in RPMI‐1640 medium (Hyclone). All culture media were supplemented with 10% fetal bovine serum (FBS, Gibco) and 1% penicillin–streptomycin. Cells were cultured at 37°C and 5% CO_2_. Transient transfection with small interfering RNA (siRNA) was performed to silence DDX21 and TRIM28. The transfection reagent was obtained from Yeasen Biotechnology, and the siRNA oligonucleotides were synthesized by General Biosystems.

### 2.13 Western blotting

Cell lysates were prepared using 1 × SDS lysis buffer, then isolated on SDS‐PAGE and transferred onto polyvinylidene fluoride (PVDF) membranes. After blocking in 5% skimmed milk for 1 h, the PVDF membranes were incubated overnight with primary antibodies including DDX21 (YT7354, Immunoway) and TRIM28 (YM8120). Immunoway and β‐actin (4967, CST). Subsequently, the membrane was incubated with the corresponding secondary antibody at room temperature for 1 h. Then, the ECL kit (Millipore) was used as per the instructions to detect the protein bands.

### 2.14 Quantitative real‐time PCR

According to the manufacturer's instructions, quantitative real‐time PCR (qRT‐PCR) was performed using the qRT‐PCR kit (Yeasen). Total RNA was extracted using the RNA Extraction kit (TIANGEN), and reverse transcribed into cDNA using Hifair® lll 1st Strand cDNA Synthesis perMix for qPCR (gDNA digester plus). Subsequently, qPCR was performed using Hieff UNICON® Universal Blue qPCR SYBR Green Master Mix, and the relative gene expression was calculated using the 2△△Ct method. The primer sequence of PCR experiment: DDX21, R: GCCTGATGGCATCTTTGCTG, F: GCTGCACGTGGGTTAGACAT; TRIM28, R: AGGGCCTGTTGAGTTAGTGC, F: GCACTGGACCATGACCAAGA; β‐actin, R: CAGAGCAAGAGAGGCATCC, F: CTGGGGTGTTGAAGGTCTC.

### 2.15 Cell viability assay (CCK‐8)

The experiment was conducted according to the actual instructions. In simple terms, approximately 800 cells were inoculated into a 96‐well plate. After culturing for the corresponding time, 10% CCK‐8 reagent (Biosharp) was added to each well. After incubation for 2–4 h, the absorbance (OD) of each well was detected at 450 nm.

### 2.16 Colony formation test

Approximately 150 cells were placed in each well of a 12‐well plate and kept in a 5% CO_2_ incubator at 37°C. Fourteen days later, the colonies were fixed with 4% paraformaldehyde for 10 min, and then stained with 0.4% crystal violet (G1070, Solarbio) for another 20 min. Then the cells were washed three times with phosphate buffer saline, and the colonies were counted for statistical analysis.

### 2.17 Transwell assay

Tumor cells were resuspended in serum‐free medium with a concentration of 1.2 × 10^5^/mL, and 200 μL of the suspension was added to the upper compartment of the Transwell insert (FALCON). Place 500 μL of complete culture medium in the lower well. After 24 h, the cells were fixed with 4% paraformaldehyde for 10 min, and then stained with 0.4% crystal violet (#G1070 Solarbio) staining solution for 20 min. Observe and count the number of migrating cells under a light microscope for statistics.

### 2.18 Wound healing assay

Cells were grown to a density of ≥90% confluence. A sterile 200 μL pipette tip was used to create vertical scratches on the cell monolayer. Fresh serum‐free medium was then added, and images were taken immediately (0 h). The cells were subsequently cultured under standard incubation conditions (37°C, 5% CO_2_) for 24 h, and photographs were taken again to assess wound closure.

### 2.19 Statistical analysis

Statistical analysis for this study was performed using R software (version 4.4.1) and GraphPad Prism (version 9.0). Randomization and blinding are not required. For comparison of differences between binary and continuous variables, we used the Student's *t*‐test. *p* < 0.05 was considered statistically significant.

## 3 RESULTS

### 3.1 Differential expression analysis in CRC

We first analyzed RNA‐seq data from 702 CRC patients in the TCGA dataset. Differential analysis between cancer and normal samples was conducted using the “limma” package (|log_2_FC| ≥ 1, *p* < 0.05), which identified 3671 genes, including 2486 upregulated and 1185 downregulated (Figure S1A,B, Table S2). Next, GO, KEGG and GSEA pathway enrichment analyses were used to examine the DEGs, which were mainly enriched in biological functions related to the cell cycle, cytokines, and fat metabolism (Figure S1C–F).

### 3.2 Construction of the risk model of LLPS‐related genes in CRC

**FIGURE 1:**
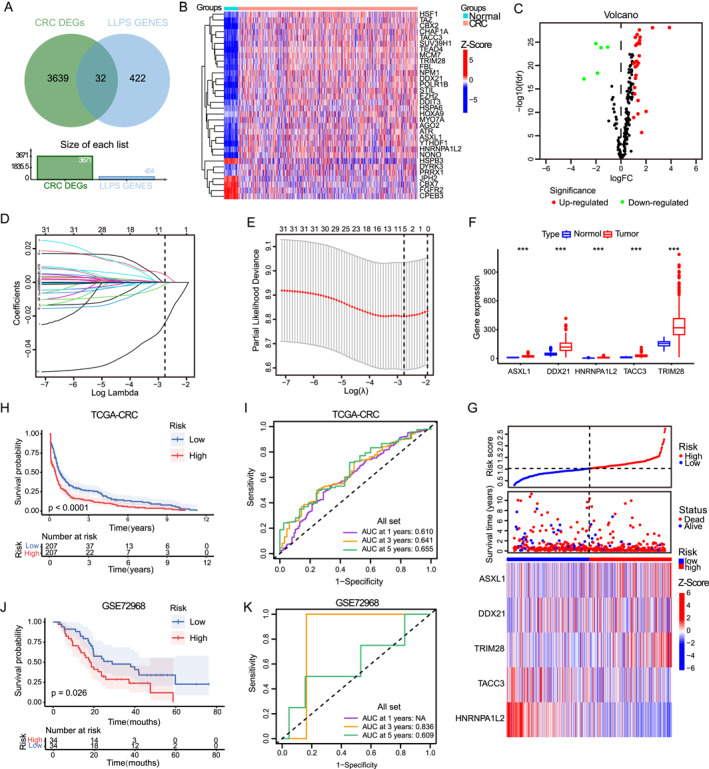
Construction of the risk model of LLPS‐related genes in CRC. (A) Venn diagram shows 32 overlaps of CRC genes and LLPS‐related genes. (B, C) Heat maps (B) and volcano maps (C) show the expression of LLPS‐related genes in the CRC cohort. (D) The coefficient distribution of the five key‐related genes of LLPS selected by LASSO Cox regression analysis. (E) Select the optimal parameters in the LASSO Cox regression analysis and conduct 10‐times cross‐validation. (F) Expression analysis of five key‐related genes of LLPS in the CRC cohort. (G) Risk scores, survival distributions, and expression heat maps of five key‐related genes of LLPS. (H) Kaplan–Meier analysis of survival differences between the high‐ and low‐risk groups in the CRC cohort. (I) ROC curves of 1‐, 3‐, and 5‐year risk scores in the CRC cohort, (J) Kaplan–Meier analysis for verifying the survival differences between the high‐ and low‐risk groups in the dataset. (K) Verify the ROC curves of the 1‐, 3‐, and 5‐year risk scores in the dataset. CRC, colorectal cancer; LLPS, liquid–liquid phase separation; ROC, receiver operating characteristic. *p* < 0.05 indicates a statistically significant difference. (***p < 0.001).

To identify the LLPS‐DEGs in CRC, we crossed the above‐mentioned DEGs with 454 phase separation‐related genes in the PhaSepDB, obtaining 32 genes, including 27 upregulated and 5 downregulated genes (Figure 1A–C, Tables S3 and S4). Next, we included 32 LLPS‐DEGs in the LASSO Cox regression model. After the screening, 5 genes remained with nonzero regression coefficient, including ASXL1, DDX21, HNRNPA1L2, TACC3, and TRIM28 (Figure 1D,E). The results of the CRC cohort showed that in CRC tissues, all five key genes were in a highly expressed state (Figure 1F). Furthermore, we combined the *λ* optimal value of LASSO Cox regression analysis with five key genes to construct the risk characteristics of LLPS‐related genes. Calculate the risk score of each patient according to the following formula: Risk score = 4.550809e‐03 × ASXL1 expression + 1.096763e‐05 × DDX21 expression + (−2.406514e‐02) × HNRNPA1L2 expression + (−1.111602e‐03) × TACC3 expression + 1.326908e‐04 × TRIM28 expression. The median risk score value was used to divide patients into the high‐ and the low‐risk groups. The integration of prognosis‐related LLPS gene expression with risk stratification facilitated the construction of a heatmap, which illustrated the survival profiles and LLPS gene expression patterns of high‐ and low‐risk CRC patients (Figure 1G). To evaluate the prognostic performance of the model, KM analysis was conducted. The higher the risk score of CRC patients, the lower the OS rate of the patients (Figure 1H). To further verify the accuracy of the model prediction, the ROC analysis results showed that the model had a strong ability to predict the 1‐, 3‐ and 5‐year survival rates of CRC patients (Figure 1I). These findings established five key LLPS‐related genes as unique prognostic indicators for CRC patients.

We conducted a similar scoring of the risk score prediction model for each patient in the validation set cohort GSE72968 and divided them into the high‐ and low‐risk groups based on the median risk score. When combined with survival data, patients in the high‐risk group exhibited significantly shorter OS compared with those in the low‐risk group (Figure 1J). The area under curve (AUC) values predicted by the ROC curve at 3 and 5 years were 0.836 and 0.609, respectively (Figure 1K). To sum up, the results of CRC and the validation cohort consistently indicate that the risk characteristics of LLPS‐related genes we constructed have a strong ability and accuracy in predicting the prognosis of CRC patients.

### 3.3 Development and verification of nomogram

**FIGURE 2:**
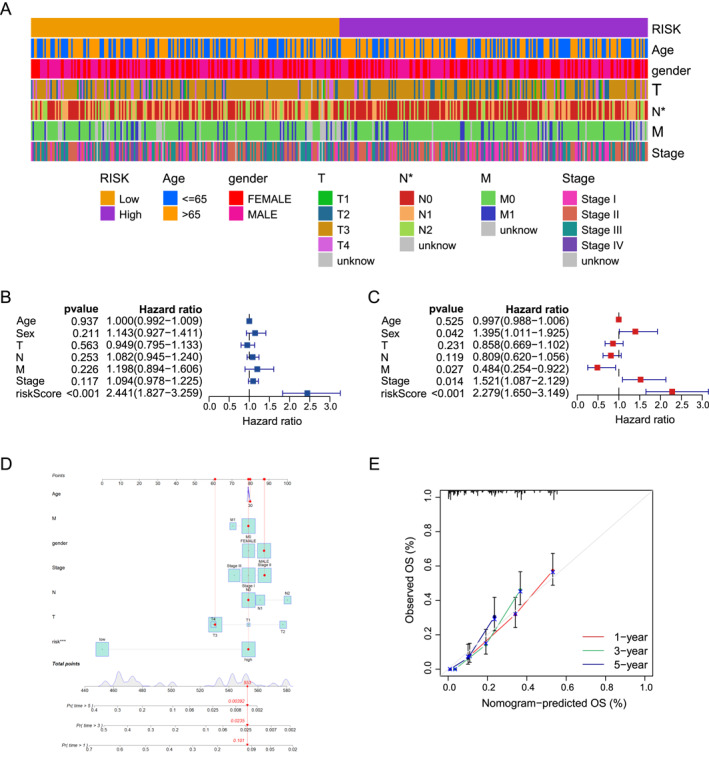
Development and verification of nomogram. (A) Heat map shows the distribution of clinicopathological characteristics in high‐ and low‐risk groups. (B) Forest plot shows the univariate Cox regression analysis of clinicopathological parameters and OS. (C) Forest plot shows the multivariate Cox regression analysis of clinicopathological parameters and OS. (D) Nomogram evaluates the overall survival of CRC patients at 1, 3, and 5 years; (E) Prognostic calibration plot verifies the differences between the predicted probabilities and the actual probabilities of the model at different time points. CRC, colorectal cancer; OS, overall survival.

To examine the prognostic value of the model, the clinical‐relevant heat map showed the distribution of clinicopathological features in the high‐ and low‐risk groups. We found that the metastasis rate of high‐risk CRC patients was higher (Figure 2A). Through univariate COX analysis, we found that only LLPS was a factor affecting the prognosis of CRC (*p* = 0.001, HR = 2.441, 95% CI 1.827−3.259) (Figure 2B). However, multivariate COX analysis showed that Sex, M, Stage, and LLPS were all independent prognostic factors (*p* = 0.001, HR = 2.279, 95% CI 1.650−3.149) (Figure 2C). These results indicate that the five key genes are reliable predictors of the prognosis of CRC patients, and are not related to other clinicopathological characteristics, including histological grade, gender, age, and pathological stage. To enhance the clinical applicability of the risk model, a nomogram was constructed for CRC patients incorporating gender, age, clinical stage, and risk score (Figure 2D), which accurately predicted 1‐, 3‐, and 5‐year OS. The calibration plots further demonstrated that the predicted survival probabilities closely approximated the actual 1‐, 3‐, and 5‐year survival outcomes (Figure 2E).

### 3.4 Association of five risk genes with disease subtypes

To clarify the association between the five LLPS‐driven genes and CRC subtypes, we first examined their expression in colon and rectal cancers according to anatomical classification. The results showed that only ASXL1 had expression differences in colon cancer and rectal cancer, whereas the expression of the other four genes did not show significant differences in the two cancers (Figure S2A). KM analysis showed that the prognostic performance was consistent in both models. The higher the risk score of the patients, the lower the OS rate (Figure S2B,C). These analysis results indicate that the LLPS driver genes we identified are randomly distributed in colon cancer and rectal cancer. Secondly, the most recognized disease classification of CRC at present is the CMS classification proposed by Guinney et al. in 2015. The CMS classification is an important milestone in the research of CRC. It classifies this disease into four significantly different subtypes at the molecular level: CMS1 (Immune type), CMS2 (Classical type), CMS3 (Metabolic type), and CMS4 (Interstitial type).^17^ We used the R package “CMSclassifier” to classify TCGA CRC data by CMS (CMS1 13%, CMS2 64%, CMS3 10%, and CMS4 13%), and analyzed the expression of five genes in four CMS classifications. We found that ASXL1, DDX21, and HNRNPA1L2 were significantly highly expressed in CMS2 and CMS4, TACC3 was significantly highly expressed in CMS1, whereas TRIM28 was basically expressed consistently in the four types (Figure S2D–H). KM analysis showed that only in CMS2, the higher the risk score of CRC patients, the lower the OS rate of patients, which was statistically significant (Figure S2I–L). These analysis results indicate that the LLPS process may have significant subtype specificity in CRC and a strong prognostic predictive ability in the CMS2 subtype. Our findings indicate that LLPS exhibits significant subtype specificity in CRC and possesses strong prognostic value within the CMS2 subtype. This suggests that LLPS‐driven biological processes are fundamental to the malignant progression of CMS2 tumors. Consequently, targeting key LLPS‐related genes may represent a particularly promising therapeutic strategy for CMS2 patient cohort.

### 3.5 Enhanced performance of the risk model compared to unsupervised clustering

**FIGURE 3:**
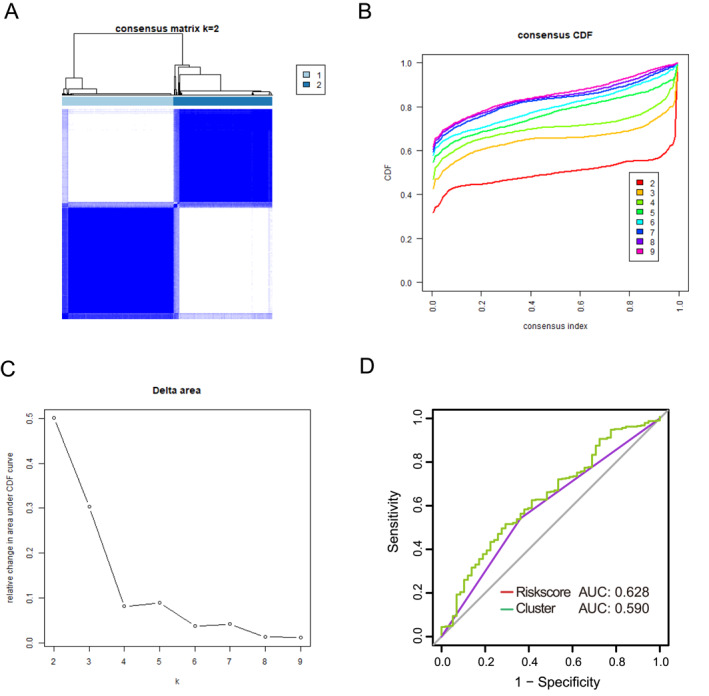
Enhanced performance of the risk model compared to unsupervised clustering. (A) Unsupervised consensus clustering analysis of CRC when the number of clusters *k* = 2; (B) CDF when *k* = 2–9; (C) Relative change of the area under the CDF curve when *k* = 2–9. (D) ROC curves show the prognostic predictive performance of the LLPS risk model and the clustering method of 32 differentially expressed genes. CDF, cumulative distribution function; CRC, colorectal cancer; LLPS, liquid–liquid phase separation; ROC, receiver operating characteristic.

To further verify the performance of the risk model, we adopted another approach and analyzed 32 LLPS‐DEGs without any additional screening. Unsupervised clustering shows two different clusters, namely C1 and C2 (Figure 3A–C). Furthermore, we found that the AUC under the ROC curve of the LLPS risk model was 0.628, which was superior to the AUC of 0.59 of the Cluster model (Figure 3D). The above results once again demonstrate that the risk characteristics of LLPS‐related genes we constructed have higher accuracy in predicting the prognosis of CRC patients.

### 3.6 Tumor mutation burden analysis and functional enrichment analysis based on the risk model

**FIGURE 4:**
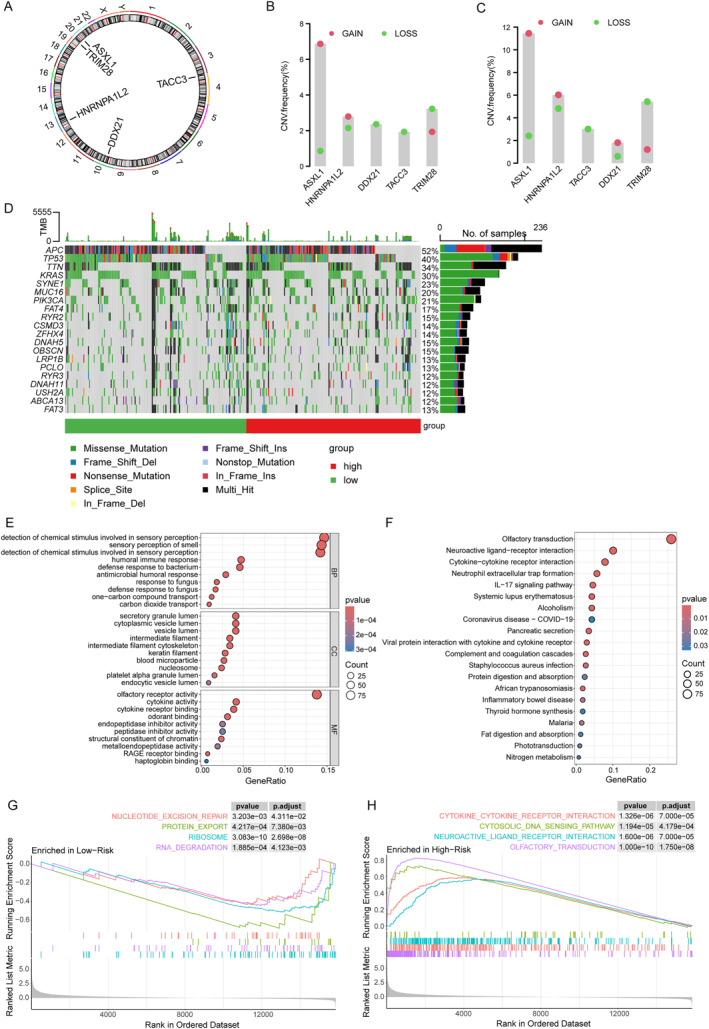
Tumor mutation burden analysis and functional enrichment analysis based on the risk model. (A) The positions of five key genes in the chromosome. (B, C) In colon cancer (B) and rectal cancer (C), CNV mutations are widely present in five prognostic LLPS genes. (D) Waterfall plot shows the top 20 genes with the highest mutation frequencies in both the high and low‐risk groups. (E) GO enrichment analysis of differentially expressed genes in the high‐ and low‐risk groups. (F) KEGG enrichment analysis of differentially expressed genes in the high‐ and low‐risk groups. (G, H) GSEA analyzed the potential enrichment pathways of the low‐risk group (G) and the high‐risk group (H). LLPS, liquid–liquid phase separation. CNV, copy—number variant; GO, gene ontology; GSEA, gene sete enrichment analysis; KEGG, kyoto encyclopedia of genes and genomes.

We downloaded the nucleotide mutation data of CRC from TCGA to explore the differences in genomic mutations between the two risk groups. Firstly, we presented the locations of five key prognostic genes on the chromosome (Figure 4A). Next, the copy—number variant (CNV) of five key prognostic genes in CRC was analyzed. The results showed that ASXL1 and HNRNPA1L2 had high amplification rates, whereas TACC3 and TRIM28 had high CNV deletions (Figure 4B,C). Additionally, characterization of somatic mutations identified APC, TP53, TTN, KRAS, and SYNE1 as the five most frequently mutated genes. The mutation frequencies of these genes were notably higher in the high‐risk group compared to the low‐risk group (Figure 4D). In order to study the changes in related biological processes and pathways between the two risk groups. We conducted GO, KEGG, and GSEA enrichment analyses on the DEGs in the low‐risk and low‐risk groups. The results showed that the functions of the DEGs in different groups were involved in multiple signaling pathways. In GO analysis, they are mainly involved in immune, metabolic and cytoskeletal or chromatin structured processes, such as humoral immune responses, transport of one‐carbon compounds and carbon dioxide, intermediate fibers, nucleosomes and other processes (Figure 4E). KEGG enrichment analysis revealed participation in some classical pathways, primarily in immune and inflammatory signaling pathways, such as cytokine–cytokine receptor interactions, IL‐17 signaling pathways, and complement and coagulation cascade reactions; Secondly, there are metabolic and digestive pathways, such as fat digestion and absorption and nitrogen metabolism (Figure 4F). Furthermore, GSEA enrichment analysis revealed that the low‐risk group might enrich DNA repair and immune pathways, which was consistent with a better prognosis (Figure 4G,H).

### 3.7 Tumor immune microenvironment analysis and drug sensitivity analysis based on risk models

**FIGURE 5:**
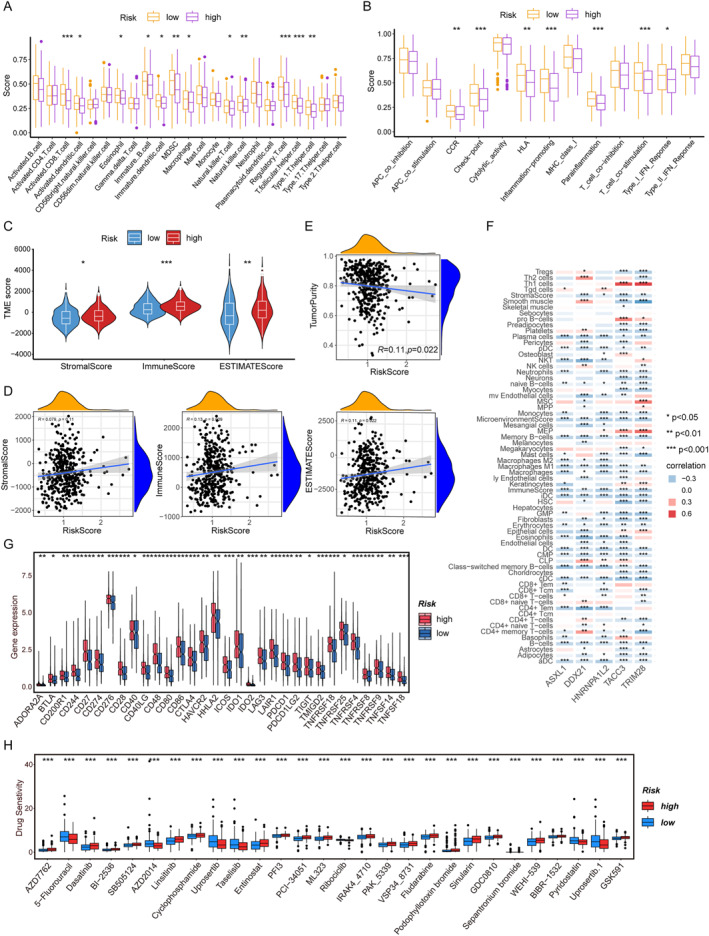
Tumor immune microenvironment analysis and drug sensitivity analysis based on risk models. (A) The differences in immune cell infiltration between the high‐ and low‐risk groups. (B) The differences in immune‐related functions between the two risk groups. (C) The differences in immune scores, matrix scores, and estimated scores between the two risk groups. (D) Scatter plot shows the correlation analysis between the risk score and the purity of tumor cells. (E) Scatter plots show the correlation analysis between the risk score and the immune score, the matrix score, and the estimated score. (F) Correlation analysis of key genes and immune cells. (G) Differences in immune checkpoints between the two risk groups. (H) The difference in drug sensitivity between the two risk groups was expressed as log (IC50). *p* < 0.05 indicates a statistically significant difference. (*p < 0.05; **p < 0.01; ***p < 0.001).

Enrichment analysis revealed that the enrichment was mainly in the immune and inflammatory responses. To verify this result, we conducted immune infiltration analysis. Firstly, we found that the proportions of 23 types of immune cells were quite abundant. Among them, the expression levels of activated CD8 T cells, bone marrow derived suppressor cells, natural killer (NK) cells, regulatory T cells, Tfh cells and Th1 helper cells in the high‐risk group were all lower than those in the low‐risk group. The decreased content of these cells indicates that immune infiltration may affect the prognosis of patients (Figure 5A). The quantitative enrichment scores indicate that the prognosis of high‐risk group may also be affected by reduced immune function, including functions such as immune checkpoints, pro‐inflammatory responses, accessory inflammation, and T‐cell co‐stimulation (Figure 5B). Furthermore, the high‐risk group exhibited significantly higher ImmuneScores, StromalScores, and ESTIMATEScores compared to the low‐risk group (*p* < 0.05) (Figure 5C). Consistent with this finding, correlation analysis revealed that the risk score was positively associated with the ImmuneScore, StromalScore, and ESTIMATEScore, but negatively associated with tumor purity (Figure 5D,E). The above results further prove the result of immune infiltration. Furthermore, the heat map shows the correlations between the five genes involved in constructing the risk model and various immune cells (Figure 5F). Based on the above results, we further analyzed the differences in immune checkpoints between the high‐ and low‐risk groups. The results showed that most factors were upregulated in the high‐risk group, and these patients might benefit from immune checkpoint inhibitors (ICIs) (Figure 5G).

To study the relationship between patients in the high‐ and low‐risk groups and the sensitivity to anticancer drugs, we calculated the IC50 of anticancer drugs in the two groups. The results showed that the high‐risk group exhibited a higher predictive sensitivity to Entinostat and a lower predictive sensitivity to 5‐Fu, indicating that the high‐risk group might benefit more from 5‐Fu treatment. Conversely, patients with low‐risk group may be more sensitive to Entinostat treatment (Figure 5H).

### 3.8 Phase separation of five key predictive genes

**FIGURE 6:**
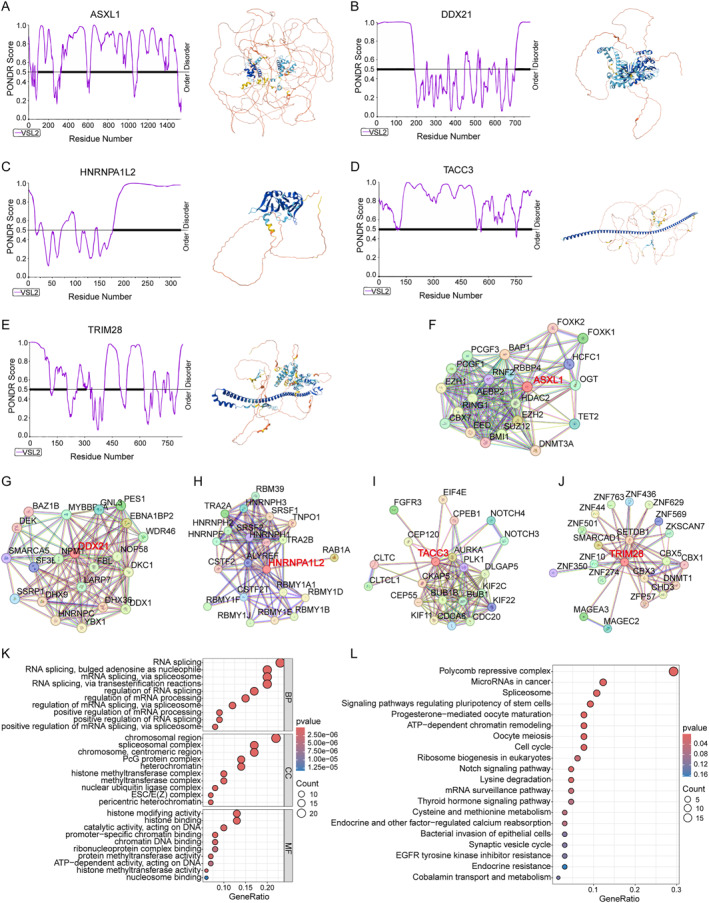
Phase separation of five key predictive genes. (A–E) The intrinsic IDR diagrams and protein structure diagrams of the key genes ASXL1 (A), DDX21 (B), HNRNPA1L2 (C), TACC3 (D), and TRIM28 (E) calculated by the PONDR website. Those with PONDR scores higher than 0.5 are disordered regions, and those with scores lower than 0.5 are ordered regions. In the protein structure, the blue area is the ordered region and the orange area is the disordered region. (F–J) PPI networks of key genes ASXL1 (F), DDX21 (G), HNRNPA1L2 (H), TACC3 (I), and TRIM28 (J) obtained from the STRING database. (K) GO enrichment analysis of interacting proteins predicted by five key genes. (L) KEGG enrichment analysis of interacting proteins predicted by five key genes. GO, gene ontology; KEGG, kyoto encyclopedia of genes and genomes; PPI, protein‐protein interaction.

To study the structural characteristics of five key predictive proteins, we used the PONDR site to predict the disordered regions of the proteins and found that all five key predictive proteins had strong intrinsic disordered regions (IDRs), indicating a strong phase separation ability (Figure 6A–E). Subsequently, we obtained the protein‐protein interaction (PPI) networks of the top 20 proteins interacting with each key protein from the STRING database (Figure 6F–J), and conducted GO and KEGG enrichment analyses using these interacting proteins. The results of GO enrichment analysis showed that the focus was on the biological processes, cellular components and molecular functions related to RNA splicing and chromatin modification. This result suggests that the five key proteins and interacting proteins may undergo common phase separation through interaction, forming dynamic membraneless organelles that play a crucial role in the cross‐network of RNA splicing and epigenetic regulation, thereby altering the function of the cell. The results of KEGG enrichment analysis indicated that the five key proteins might act as “multifunctional hubs” within cells, dynamically integrating the three key biological processes of epigenetic regulation, RNA processing, and metabolic reprogramming through phase separation mechanisms. Abnormal phase separation may lead to a vicious cycle of epigenetic disorders, splicing dysregulation, and metabolic reprogramming, promoting the occurrence of diseases (Figure 6K,L). This discovery provides a new perspective for understanding how cells coordinate regulatory networks at different levels and lays a theoretical foundation for developing novel therapeutic strategies targeting phase separation interfaces.

### 3.9 Cellular localization of risk model genes

**FIGURE 7:**
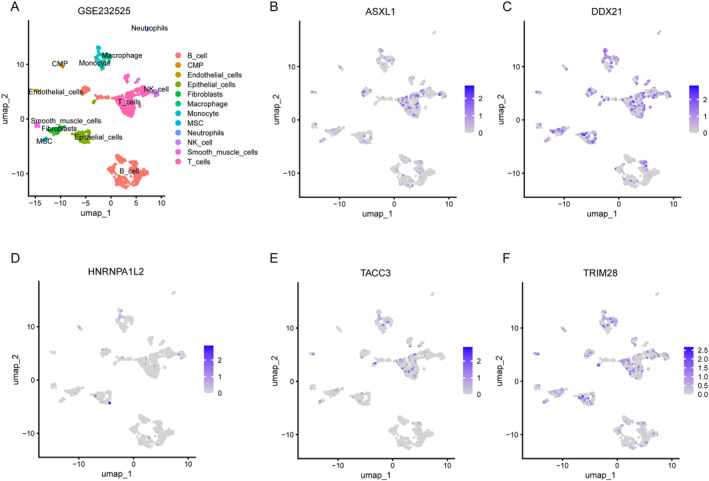
Cellular localization of risk model genes. (A) Use Umap to shrink the data of tumor tissues and annotate each cluster; (B–F) The expression of ASXL1 (B), DDX21 (C), HNRNPA1L2 (D), TACC3 (E), and TRIM28 (F) at the single‐cell level.

To further explore these five key genes, we also conducted single‐cell analyses to study their expression and distribution in different cells. The analysis of the GSE232525 database indicates that there are significant differences in the distribution of five genes in the tumor tissues (Figure 7A). We found that ASXL1 and TACC3 are mainly expressed in T cells, suggesting that they may be involved in the remodeling of the immune microenvironment of CRC (Figure 7B,E). T cell infiltration is an important indicator of the prognosis of CRC. The high expression of these two genes may affect the tumor immune escape mechanism by regulating T cell activation or migration. DDX21 and TRIM28 are expressed in almost all cell types (Figure 7C,F). It is worth noting that TRIM28 reportedly exhibits a dual function in CRC, where it promotes tumor cell proliferation while concurrently inhibiting epithelial–mesenchymal transition (EMT) via epigenetic regulation. In contrast, HNRNPA1L2 is expressed at low levels in epithelial cells and may be involved in maintaining their differentiated state (Figure 7D).

### 3.10 Identifying the oncogenic role of DDX21 and TRIM28 in CRC

**FIGURE 8:**
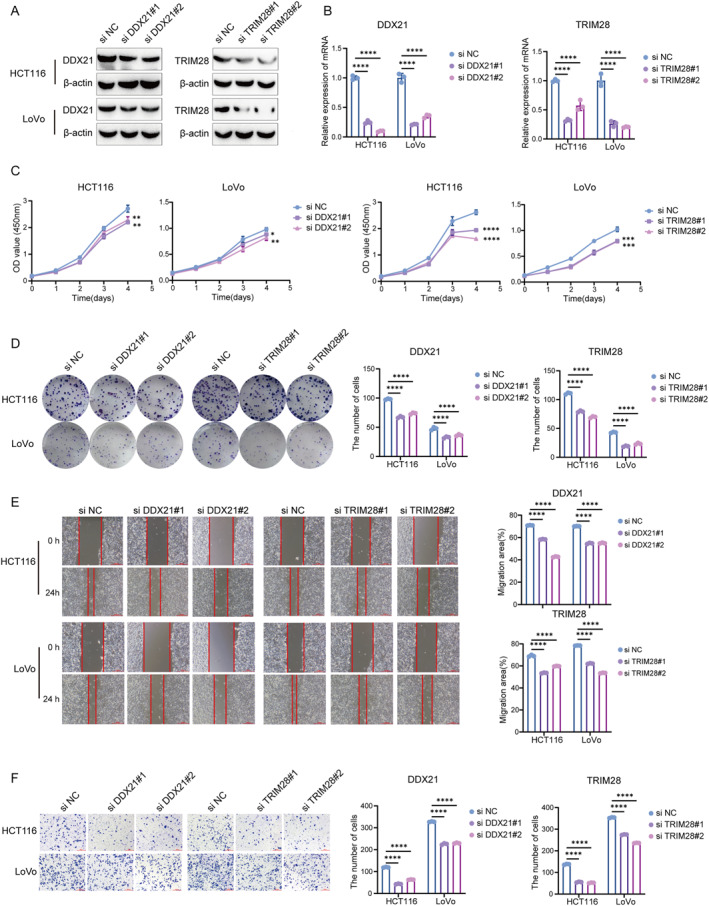
DDX21 and TRIM28 promote the proliferation and migration of CRC cells. (A, B) Western blot (A) and PCR (B) were used to detect the knockdown of DDX21 and TRIM28 in HCT116 and LoVo cells. (C, D) CCK‐8 (C) and colony formation (D) assays were used to detect the changes in the proliferation ability of HCT116 and LoVo cells after knockdown of DDX21 or TRIM28. (E, F) Wound healing assays (E) and transwell (F) were used to detect the changes in the migration ability of CRC cells after knockdown of DDX21 or TRIM28. Scale bar: 200 μm. *n* = 3, one‐way analysis of variance was used for analysis. *p* < 0.05 indicates a statistically significant difference. (*p < 0.05; **p < 0.01, ***p < 0.001, ****p < 0.0001). CRC, colorectal cancer.

Our bioinformatics analysis revealed that the expression levels of DDX21 and TRIM28 in tumor tissues were elevated compared with normal tissues, and they were classified as high‐risk prognostic factors. To better verify the functional roles of DDX21 and TRIM28 in the progression of CRC, we conducted in vitro cytological experiments on the silencing of DDX21 and TRIM28. Firstly, we examined the expression levels of DDX21 and TRIM28 in both normal cell lines and knockdown cell lines. The results showed that the expressions of DDX21 and TRIM28 in the knockdown group were downregulated (Figure 8A,B). In the CCK‐8 and cell colony formation experiments, after knocking down DDX21 or TRIM28, the proliferation ability of cells was significantly inhibited (Figure 8C,D). Wound healing and transwell experiments demonstrated that the migration ability of cells was significantly reduced after knocking down DDX21 or TRIM28 (Figure 8E,F). In conclusion, DDX21 and TRIM28 play a crucial role in the progress of CRC.

## 4 DISCUSSION

CRC is the second leading cause of death worldwide and is an increasingly serious global health problem.^18^, ^19^ In recent years, it has been found that the incidence of CRC is constantly increasing, and the age of onset is getting younger and younger.^20^ The standard treatments for CRC include surgery, chemotherapy, radiotherapy, molecular targeted therapy, and immunotherapy. Although most CRC patients initially respond to the treatment, the overall response rate of first‐line treatment varies greatly.^21^, ^22^ This is because CRC is highly heterogeneous. Even within the same tumor stage, there are differences in the results.^23^ The complexity of the mechanism of cancer occurrence and development makes tumor treatment difficult.

In recent years, the discovery of LLPS has provided a new direction for the clarification of tumor treatment and deserves more in‐depth research. LLPS was originally defined and studied in the field of engineering as a physicochemical phenomenon.^24^ In recent years, a growing body of research has highlighted the fundamental importance of LLPS in both cell biology and oncology.^25^, ^26^, ^27^ Studies have shown that LLPS are an important molecular mechanism for the formation of membrane less organelles within cells.^28^ Under normal physiological conditions, LLPS participate in and coordinate a variety of key cellular biological processes through precise spatiotemporal regulation, including but not limited to gene transcriptional regulation, protein homeostasis maintenance, and DNA damage repair.^13^, ^29^, ^30^, ^31^, ^32^ During the process of tumorigenesis and development, the abnormal regulation of LLPS may lead to the formation of carcinogenic biomolecular aggregates, and then promote tumor progression by activating abnormal signaling pathways or altering extracellular matrix interactions, etc.^33^, ^34^, ^35^, ^36^

In this study, we identified 3671 DEGs in CRC, among which 32 were related to LLPS. Through LASSO Cox regression analysis, we further refined to five key LLPS‐related genes (ASXL1, DDX21, HNRNPA1L2, TACC3, and TRIM28) that affect the prognosis of CRC. A prognostic model based on five genes was subsequently constructed to assign a risk score to each patient. Stratification using this score revealed that patients in the high‐risk group had a significantly worse prognosis. Our risk model demonstrated robust predictive accuracy for 1‐, 3‐, and 5‐year survival rates in both the training and validation cohorts, highlighting its potential clinical application value and providing a reference for the prognostic assessment of CRC.

Among our five key genes, ASXL1 and TRIM28 are known epigenetic regulatory factors, respectively.^37^, ^38^ ASXL1 mutations are associated with poor prognosis of myeloid malignancies, and their overexpression in CRC may similarly drive epigenetic instability.^39^ TRIM28 is a transcriptional co‐suppressor and is associated with tumor suppression and carcinogenicity in various cancers.^40^ DDX21 is an RNA helicase involved in the biogenesis of ribosomes and the formation of stress granules.^41^, ^42^ Its overexpression in CRC may promote protein synthesis and support the rapid growth of tumors. TACC3 is a centrosomal protein that regulates the stability of the mitotic spindle, and its dysregulation is related to chromosomal instability in cancer.^43^, ^44^ HNRNPA1L2 is an RNA‐binding protein that has been less studied and may lead to selective splicing dysregulation.^45^ This result provides new therapeutic targets and strategies for the treatment of CRC.

In addition, this study also delved deeply into the association between five LLPS driver genes and the molecular typing of CRC, revealing their significant subtype specificity and prognostic value. It is worth noting that these genes are randomly distributed in the traditional anatomical staging (colon cancer and rectal cancer), but they show clear expression patterns in the CMS typing that reflects intrinsic molecular heterogeneity. ASXL1, DDX21, and HNRNPA1L2 are significantly highly expressed in CMS2 (classic type) and CMS4 (mesenchymal type), suggesting that they may co‐participate in the phase separation regulation process related to the activation of the classical WNT signaling pathway (characteristic of CMS2) or EMT (characteristic of CMS4). It is worth noting that the risk score demonstrated strong independent prognostic value exclusively in the CMS2 subtype. This finding suggests that the LLPS process is a core biological mechanism driving the malignant progression of these specific tumors. CMS2, as the subtype with the highest proportion (64%), its occurrence and development are highly dependent on abnormal cell proliferation and differentiation. Our findings suggest that the key genes of LLPS may play the role of a “molecular hub” in this process. This provides a solid theoretical basis for developing precise phase separation targeted therapies for the CMS2 patient population. In the future, it is expected that by regulating the phase separation ability of these genes, tumor progression can be intervened, and patient prognosis can be improved.

Our immune infiltration analysis revealed that high‐risk CRC patients exhibited reduced infiltration of immune cells, including CD8+ T cells and NK cells, suggesting immunosuppression of the TME. This is consistent with previous studies, indicating that immune rejection leads to a poor prognosis of CRC.^46^, ^47^ It is worth noting that most immune checkpoint molecules are upregulated in high‐risk patients, indicating potential reactivity to ICIs. However, key immune‐related pathways, including those for immune checkpoints, pro‐inflammatory responses, and T‐cell co‐stimulation, were suppressed in the high‐risk group. This observation is consistent with previous findings in the literature. Such diminished immune activity, particularly the reduction in immune cell infiltration, may limit the efficacy of ICIs, suggesting that combination therapies are needed to enhance T‐cell recruitment.^48^, ^49^

In the prediction results of drug sensitivity, we found that the sensitivity of the high‐risk group to Entinostat increased, which might be due to epigenetic dysregulation driven by ASXL1 and TRIM28. On the contrary, patients with low risk respond better to 5‐FU, which might be due to the intact DNA repair mechanism. These findings emphasize the necessity of a risk‐stratified treatment strategy for CRC.

According to literature reports and website predictions, all five key genes have IDRs, which are the hallmarks of LLPS proteins. Their PPI networks show RNA splicing and chromatin modification enrichment, suggesting that these genes may form dynamic condensates that regulate transcriptional and post‐transcriptional processes. For example, DDX21 can form a co‐aggregate with splicing factors to regulate the oncogenic RNA subtype.^50^ Similarly, ASXL1 and TRIM28 can form phase‐separation hubs, recruit epigenetic modification factors, and change the chromatin accessibility of CRC.^51^, ^52^ The dynamic condensates formed by these genes can serve as potential therapeutic targets, regulating oncogenic RNA splicing and epigenetic modifications by intervening in their phase separation process. Furthermore, it provides a new perspective for understanding the heterogeneity and treatment resistance of CRC. To enhance the reliability of our research results, we conducted in vitro experiments to study the functions of DDX21 and TRIM28 in CRC. Our experiments confirmed that DDX21 and TRIM28 are highly expressed in CRC and demonstrated that downregulation of either gene significantly inhibits tumor cell proliferation and migration. This result experimentally demonstrates the role of DDX21 and TRIM28 risk genes.

## 5 CONCLUSION

In conclusion, this study constructed a prognostic risk model for CRC patients from LLPS‐related genes for the first time, and further analyzed its potential functions, immune infiltration, mutations, and drug sensitivity. It not only expanded the understanding of the molecular mechanism of CRC but also provided a new direction for the development of precision treatment strategies based on LLPS, such as drugs targeting abnormal biomolecular condensates.

## AUTHOR CONTRIBUTIONS

Hui Liu, Ziwen Chen, and Jie Hao analyzed the data, and Hui Liu, Ziyi Dong, and Yaoyang Guo wrote the paper. Haiyang Zhang, Ming Gao, and Xipeng Zhang provided research ideas and financial support. Haiyang Zhang and Minghan Qiu edited the manuscript. All the authors reviewed the manuscript. Mingqing Zhang is the guarantor of this work, has full access to all the data in the research, and is responsible for the integrity of the data and the accuracy of the data analysis.

## CONFLICT OF INTEREST STATEMENT

The authors declare no conflicts of interest.

## ETHICS STATEMENT

Not applicable.

## Supporting information

Figures S1 and S2

Tables S1–S4

## Data Availability

The data that support the findings of this study are openly available in TCGA database at https://portal.gdc.cancer.gov/, GEO database at https://www.ncbi.nlm.nih.gov/geo/, or are available from the corresponding author upon reasonable request.
