# Supplementary material for: Risk model of liquid–liquid phase separation‐related genes reveals the prognosis and tumor microenvironment characteristics of colorectal cancer
Source: J Cell Commun Signal. 2025 Nov 3;19(4):e70054. doi: 10.1002/ccs3.70054 (PMC12582976; doi:10.1002/ccs3.70054)
Supplement: Supplementary file 1 — Figures S1 and S2 [file CCS3-19-e70054-s001.docx]

**Supplementary material-1**

**Supplementary Figure 1**

**
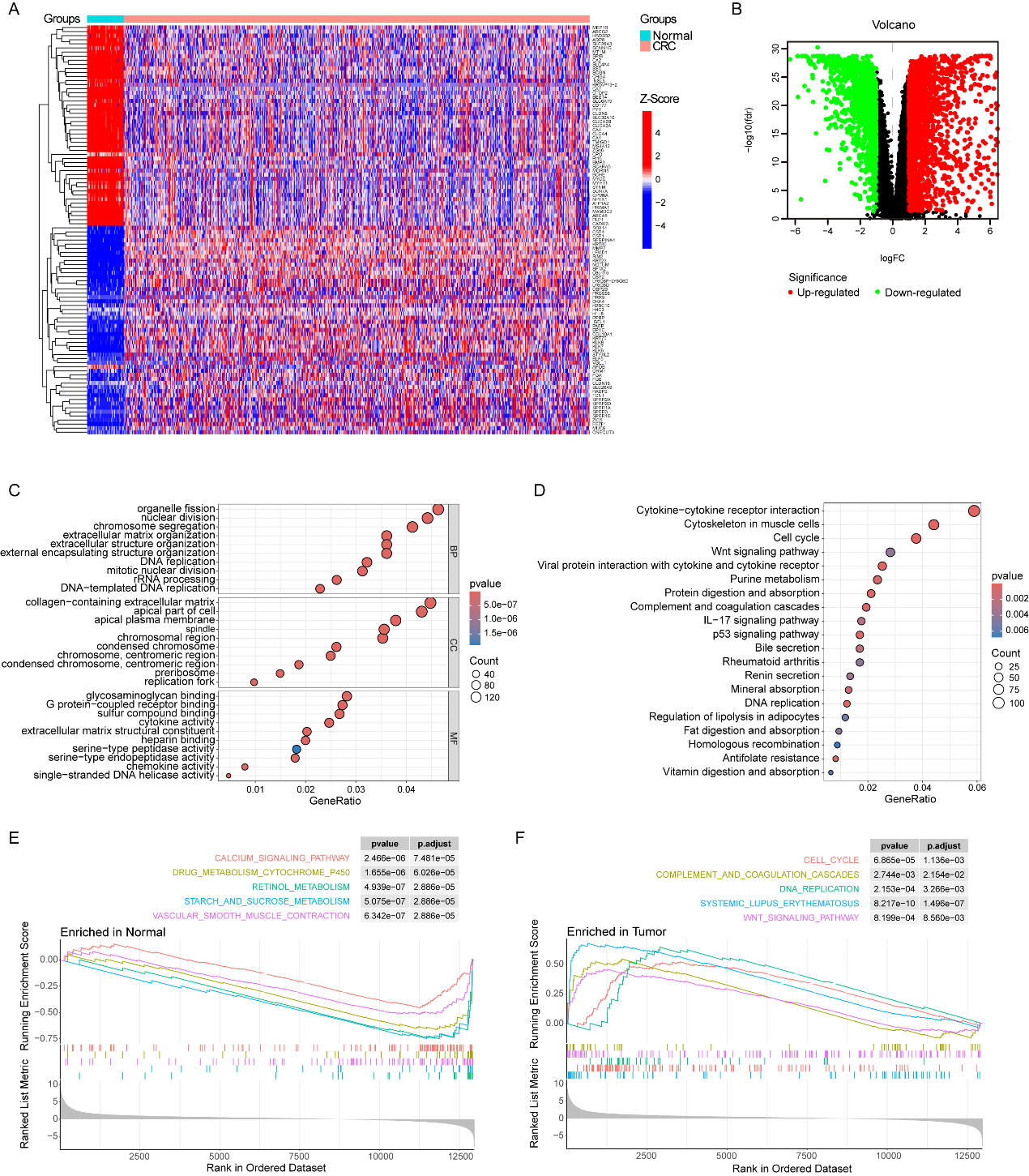
**

**Supplementary Fig.1** Differential expression analysis in CRC. **A, B.** Heat maps (**A**) and volcano maps (**B**) show the expression of differentially genes in the CRC cohort; **C.** GO enrichment analysis of differentially expressed genes; **D.** KEGG enrichment analysis of differentially expressed genes; **E, F.** GSEA analyzed the potential enrichment pathways of the normal group (**E**) and the CRC group (**F**).

**Supplementary Figure 2**


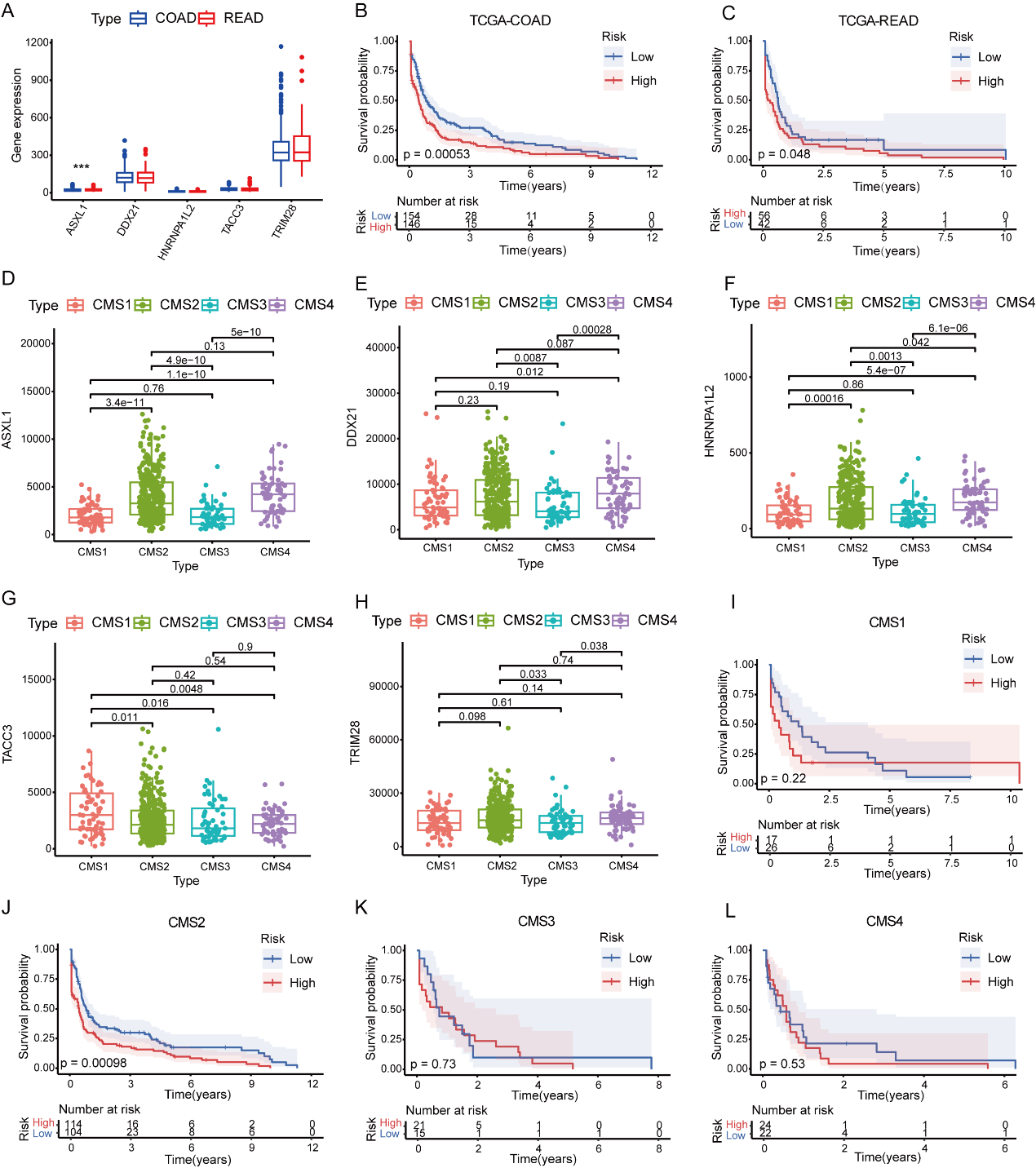


**Supplementary Fig.2** Association of five risk genes with disease subtypes **A.** Expression analysis of five key genes related to LLPS in colon cancer and rectal cancer; **B, C.** Kaplan-Meier analysis of survival differences between the high- and low-risk groups in the classification of colon cancer (**B**) and rectal cancer (**C**); **D-H.** Expression analysis of five key genes related to LLPS in the four CMS classifications; **I-L.** Kaplan-Meier analysis of survival differences between the high- and low-risk groups in the four CMS classifications.
